# Seroprevalence of bovine viral diarrhea virus (BVDV) in cattle in the Northern, Northcentral, Central, and Southern provinces of Sri Lanka

**DOI:** 10.3389/fvets.2025.1730906

**Published:** 2025-12-16

**Authors:** W. M. G. K. Weerasekara, W. M. C. Dhananjaya, W. M. D. Ashinika, V. Wijewardana, R. T. Kangethe, A. W. Kalupahana, T. A. N. Mahakapuge

**Affiliations:** 1 Department of Veterinary Pathobiology, Faculty of Veterinary Medicine and Animal Science, University of Peradeniya, Peradeniya, Sri Lanka; 2 Animal production and Health Laboratory, Joint FAO/IAEA Centre of Nuclear Techniques in Food and Agriculture, Department of Nuclear Sciences and Applications, International Atomic Energy Agency, IAEA Laboratories, Seibersdorf, Austria

**Keywords:** bovine viral diarrhea, agro-climatic zones, indirect ELISA, antibodies, seroprevalance

## Abstract

Bovine Viral Diarrhea Virus (BVDV) is a highly significant cattle pathogen with economic impacts that lead to reproductive failures, immunosuppression, and low productivity. This research paper examined the seroprevalence of BVDV in four Provinces in Sri Lanka, namely Northern, North Central, Central and Southern, which represents both wet and dry agro-climatic zones. In total, 178 archived bovine serum samples collected during 2022 and 2023 were tested to evaluate BVDV-specific antibodies with an indirect ELISA. Numerically, the wet zone had 14.7% seropositive (10/68), and none of the cattle in the dry zone were found to be positive (0/110). The prevalence may be higher in the wet zone due to grazing practices and shared water sources, which facilitate viral transmission. This paper highlights the need of constant surveillance to monitor the seroprevalence while establishing methods to detect BVDV antigens among the local cattle populations. The importance of maintaining vaccine records are needed to prevent the interference with surveillance studies. Further research with larger and more geographically diverse sampling, including buffaloes, are recommended to clarify the national status and economic impact of BVDV infection in Sri Lanka.

## 1 Introduction

Bovine Viral Diarrhea (BVD) affects cattle globally and causes significant economic losses in both the dairy and beef industries (1). Belonging to the *Pestivirus* genus within the *Flaviviridae* family, BVDV is a highly versatile and an adaptive virus. Bovine Viral Diarrhea Virus (BVDV) is classified into two main genotypes, BVDV-1 and BVDV-2. Each genotype can exist in one of two biotypes: cytopathic (CP), which causes visible damage to cells, and non-cytopathic (NCP), which does not. The NCP biotype is of particular importance, as it is more frequently linked to the development of persistent infections in cattle (2).

The infection of BVDV can manifest various clinical symptoms, which depend on the age of the infected animal, immune status of the host, and the virulence of the viral strain involved. Fever, diarrhea and immunosuppression may occur in acute infections, along with respiratory signs. This results in affected animals to be more susceptible to secondary infections (3). More severe manifestations include hemorrhagic syndrome, reproductive failures such as abortions and stillbirths, and mucosal disease, a fatal outcome in persistently infected (PI) animals (4).

Persistently infected (PI) cattle pose specific concerns as they continually serve as a reservoir of the virus and shed it frequently, which exposes other animals to infections (5). *In-utero* infections are associated with exposure to the virus in early gestation. The developing immune system then does not recognize the virus as foreign and thus, stops the PI animals from mounting an immune response against the BVDV (6). These animals are underweight and do not acquire appropriate weight for the age and later perpetuating transmission cycles within herds (7). BVDV infection in cattle can range from inapparent subclinical infection to severe clinical disease, with most herds remaining at a subclinical level that often goes unrecognized (8); however, once infection surpasses a critical threshold, the disease manifests clinically, making later detection less effective, which highlights the importance of early detection for prevention (3, 9).

Globally, BVDV have been associated with considerable economic losses brought about by decreased milk production and growth rates, increased costs for veterinary medicine, and reproductive inefficiencies in animals (10). Diseases cause losses in the infected herds through sickness and death as well as through the costs of culling PI animals. Many countries have adopted vaccination and biosecurity measures to minimize the adverse economic impact incurred by this disease to some extent (11).

The livelihoods in the rural economy of Sri Lanka are largely sustained through livestock. While BVDV is quite detrimental to the productivity and profitability of cattle farming, it brings about enormous problems in livestock production. Although BVDV is considered as a significant pathogen worldwide, with respect to Sri Lanka, the data available on this virus infection is scarce. The Epidemiology of this disease in the country is not well-understood due to a total absence of systematic surveillance and diagnosis efforts (5, 7). Furthermore, the cattle population in Sri Lanka shows clear variation across agro-climatic zones. In the wet zone (mid-country, up-country, and wet-lowland areas), exotic European breeds such as Friesian, Jersey, and Ayrshire, as well as their crosses, are widely used due to their higher milk-yield potential under semi-intensive and intensive management systems (12, 13). In contrast, in the dry lowland agro-ecological zones, cattle populations are predominantly composed of indigenous Zebu-type animals and Zebu exotic crosses, which are better adapted to heat stress, poor-quality roughage, and extensive grazing systems. Indigenous Zebu cattle in Sri Lanka have been well characterized for their genetic background and adaptive traits, including disease resistance and drought tolerance, although they typically exhibit lower milk yields compared to exotic dairy breeds (14, 15).

## 2 Materials and methods

### 2.1 Ethical approval

The research study was approved by the Ethics Committee, Faculty of Veterinary Medicine and Animal Science, University of Peradeniya (Ethical clearance ID: VERC_24_02). Consent was attained from the owners of the cattle before using the previously collected blood samples during 2022–2023.

### 2.2 Sample size determination

According to the data provided by Department of Census and Statistics-2024 (16), National Livestock Statistics, there are around 834,170 local cattle and 291,280 improved cattle. According to Orban (17) when the population size is more than 250,000 at 95% confidence level, 5% error, and the sample size will be 384. However, due to the logistical constraints, the sample size for this study was reduced to 178 cattle.

### 2.3 Sample collection

**Figure 1:**
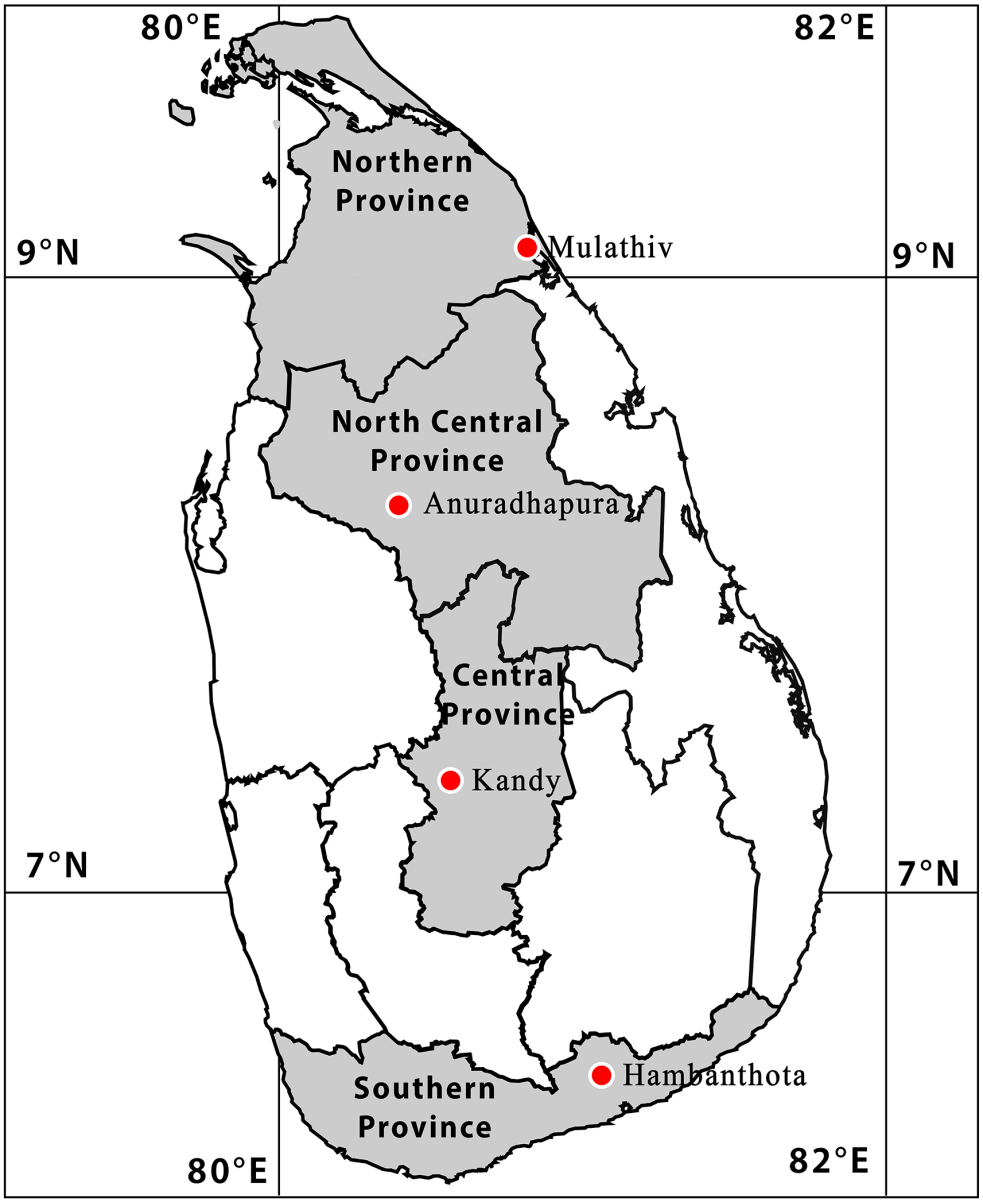
Geographical location of the study area highlighted in Sri Lankan map.

The blood samples archived from bovines were used. Those were collected from the Central Province (Kandy), Northern Province (Mullaitivu), Southern Province (Hambanthota) and North Central Province (Anuradhapura) from January 2022 to December 2023. Blood was collected from cattle of all ages, sexes, breeds and body condition score. The collected blood samples were immediately centrifuged at 3,000 rpm for 10 min to separate the serum (Figure 1). The sera were then aliquoted into sterile cryovials to prevent repeated freeze thaw cycles and stored at −20 °C until laboratory analysis. (18).

### 2.4 Detection of antibody using ELISA

The ELISA procedure to detect antibodies against BVDV was carried out using the Abnova BVDV IgG ELISA Kit (Catalog No. KA4890, Version 02), following the manufacturer’s instructions. This assay is based on a sandwich enzyme immunoassay (EIA) principle, in which wells were coated with purified and inactivated BVDV antigen to capture BVDV specific antibodies present in the test samples. After sample incubation, a horseradish peroxidase (HRP)-conjugated anti-bovine immunoglobulin was added, which binds to the captured antibodies. The presence of HRP activity is detected by a substrate solution containing 3,3′,5,5′-tetramethylbenzidine (TMB), resulting in a blue color development. Upon addition of the stop solution, the color changes to yellow, and the absorbance is measured at 450 nm.

A volume of 10 μL of each serum sample was diluted 1:20 with the sample diluent and added to the wells of the microtiter plate pre-coated with purified and inactivated BVDV antigen following manufacturer’s instructions. After mixing thoroughly, the plate was incubated at 37 °C for 60 min. Following incubation, the wells were aspirated and washed four times using a wash buffer to remove unbound components. Then, 100 μL of horseradish peroxidase (HRP)-conjugated rabbit antibodies against bovine immunoglobulin was added to each well, followed by a 30-min incubation at 37 °C. After the second washing step, 100 μL of the TMB chromogenic substrate was added to each well and incubated at room temperature for 15 min. The enzymatic reaction was then stopped by adding 100 μL of acidic stop solution, resulting in a color change from blue to yellow. The absorbance of each well was read at 450 nm using a microplate reader (TECAN, Switzerland), and the optical density (OD) values were recorded. The results were interpreted by calculating the S/P (sample to positive) ratio using the formula provided by the manufacturer:


$$\begin{array}{l} S/\text{Pratio}=\\ \;\;\frac{\text{Absorbance of the sample}}{\text{Mean absorbance of Positive Control Serum}-\text{limit}}\times 100\% \end{array}$$

Samples with S/*p* values below 30% were considered negative for BVDV-specific antibodies, those between 30 and 40% were borderline, and those equal to or above 40% were considered positive, indicating current or past exposure to BVDV. The diagnostic sensitivity and specificity of the test kit were 99.20 and 99.25%, respectively.

### 2.5 Data analysis

All obtained laboratory results were stored and cleaned in Excel Spreadsheet (Microsoft Office, 2016). Data were analyzed to determine the seroprevalence of BVDV infection in the total sampled population and at the individual herd level, following the method described by Phillips and Acheson (19):


$$\begin{array}{l} \text{Seroprevalence}\;(\%)=\\ \;\;\;\;\frac{\text{Number of animals tested seropositive}\;}{\text{Total number of animals tested}\;}\times 100\% \end{array}$$

## 3 Results

**Figure 2:**
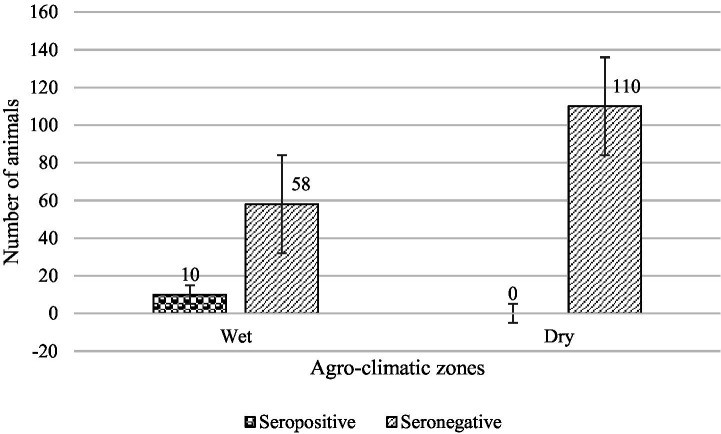
Seropositive vs. Seronegative Cattle by Agro-Climatic Zone. From the 68 cattle serum samples tested from the wet zone, 10 were seropositive for BVDV while from 110 cattle serum samples tested from dry zone were seronegative for BVDV.

The overall and individual zone seroprevalence of BVDV in cattle in Sri Lanka is summarized in Table 1. Accordingly, samples obtained from the Central Province (Kandy) represent the wet zone, whereas those collected from the Northern Province (Mullaitivu), Southern Province (Hambantota), and North Central Province (Anuradhapura) represent the dry zone. The overall seroprevalence of BVDV IgG antibodies was 5.6%. Among the two agro-climatic zones, the wet zone showed the highest seroprevalence, with 14.7% (10 out of 68 animals) testing seropositive. Notably, all the seropositive samples originated from the wet zone. In contrast, no detectable antibodies against BVDV were found among the 110 animals tested in the dry zone (0% seroprevalence). Figure 2 shows the number of seropositive and seronegative cattle in the wet and dry agro-climatic zones.

**Table 1:** Overall and individual zone seroprevalence of BVDV exposure in cattle in Sri Lanka.

Zone	Total no. of animals tested	No. of animals tested seropositive	Prevalence of BVDV exposure
Wet zone	68	10	14.7%
Dry zone	110	0	0%
Total	178	10	5.6%

## 4 Discussion

The present study provides valuable insight into the seroprevalence of BVDV in cattle across the Northern, North Central, Central, and Southern Provinces of Sri Lanka. Among the 178 animals tested, an overall seroprevalence of 5.6% was detected. BVDV antibodies were found exclusively in cattle from the wet zone (14.7%), while no seropositive cases were detected in the dry zone. It should also be acknowledged that the observed seroprevalence could vary under different herd conditions, management systems, and animal movement patterns, which were not controlled in this study. Our observed seroprevalence is high relative to the past local reports, 2.6% reported by Dasinaa (20) in the Eastern Province in which an OIE reference laboratory verified the diagnosis in the calves. Such a difference might be due to regional variation in the circulation of the virus, different diagnostic procedures (antigen or antibody detection), or temporal changes in the prevalence of the disease.

When placed in the broader South Asian context, the seroprevalence in Sri Lanka appears relatively low. For instance, Uddin et al. (18) reported a 51.1% seroprevalence in crossbred dairy cattle in Bangladesh. Raheem et al. (21) identified an overall BVDV seropositivity of 18.77% of all processed samples in Pakistan with 19.81% (129/651) of cattle and 8.82% (6/68) of buffaloes showing seroconversion. This shows a significant prevalence difference between species, which is also reflected in the trend in other countries. In India, Narayan Sarangi et al. (22) found a true prevalence of 56.67% in organised herds, where cattle had a much higher prevalence (65.42%) than buffaloes (32.49%). Tandan and Paudel (23) recorded a prevalence of 10.86% in Nepal, which is similar to our results in the wet zone, but still higher than the overall prevalence observed in Sri Lanka. Remarkably, buffalo seroprevalence in Pakistan (8.82%) is consistent with the cattle trends, in which buffaloes always have lower prevalence as compared to cattle (21, 22).

The relatively low prevalence of BVDV in Sri Lanka can be related to the differences in cattle management, biosecurity, herd composition and ecology (24, 25). Cattle farming in Sri Lanka predominantly consists of small-scale operations where farmers typically rear a few animals, and the overall animal density is relatively low compared to the larger commercial farms found in neighboring countries (26). The greater seropositivity in the wet zone might be attributed to communal grazing practices, extensive farming practices including shared feeders and waterers, abortions occurring while cattle graze, and the use of a common bulls can facilitate transmission of virus (24, 27). In the dry zone of Sri Lanka, the absence of detectable antibodies against BVDV in cattle populations may be attributed to several interactive factors. Extensive free grazing and low stocking density are prominent features of cattle management in this area, which limit close contact between animals and consequently reduce opportunities for viral transmission (25). Currently, there are no commercially available BVD vaccines widely used in Sri Lanka. Vaccination is uncommon and is limited to a small number of private farms that use imported inactivated vaccines. Among these, aluminium-salt–adjuvanted inactivated BVDV vaccines have been used on a limited basis. Since the ELISA detects both antibodies generated from the infection and vaccine, the disease prevalence rates may largely vary.

Our results support the significance of the specific surveillance and more effective biosecurity in light of the high economic cost of BVDV to cattle productivity in the form of reproductive losses, immunosuppression, and susceptibility to secondary infections. Scheduled screening of the herd, improved farm management techniques, and educational programs for the farmers are suggested to assist with the decrease of BVDV spread, especially in the regions with higher risks (18, 21, 22).

The limited sample size and geographical coverage are limitations of this study. This narrow range might not give a complete picture in terms of herd management and the agro-ecological situation in Sri Lanka. Additionally, while this study relied on antibody-based diagnostics, the inclusion of antigen detection in seropositive animals could offer a more comprehensive understanding of disease epidemiology, particularly by identifying persistently infected (PI) animals. However, clinical disease was not observed in any of the seropositive animals. Future studies should therefore include larger and more diverse cattle populations, with expanded sampling across additional provinces (23). Incorporating buffalo populations into future research would also be valuable for understanding the valid data on prevalence, transmission dynamics, and overall economic impact of BVDV in Sri Lanka (21, 22).

## 5 Conclusion

This study represents the seroprevalence of Bovine Viral Diarrhea Virus (BVDV) in cattle in Sri Lanka in the Northern, North Central, Central and Southern Provinces. An overall prevalence of 5.6% was detected, with significantly higher exposure in the wet zone (14.7%) compared to no seropositivity in the dry zone. It was not as high as in the neighboring countries, however, the extreme disparity between agro-climatic zones indicates that environmental influence and managerial practices may play a role in the dynamics of the virus dissemination. Enhancing biosecurity at the farm level, regular screening of herds, and increasing awareness among farmers are needed to reduce the spread of the BVDV and to minimize the losses linked to BVDV. The future research must involve the use of large, geographically mixed populations of cattle and buffalo herds, detection of BVDV antigen in infected herds, obtaining a complete epidemiological profile, and to direct the development of sustainable national control measures.

## Data Availability

The raw data supporting the conclusions of this article will be made available by the authors, without undue reservation.
